# Protection of human rights during involuntary treatment in mental healthcare services: A European perspective

**DOI:** 10.1192/j.eurpsy.2025.10124

**Published:** 2025-10-13

**Authors:** Martina Rojnic Kuzman, Andrea Fiorillo, Julian Beezhold

**Affiliations:** 1 Department of Psychiatry and Psychological medicine, Zagreb University Hospital Centre, Zagreb, Croatia; 2 Zagreb School of Medicine, Zagreb, Croatia; 3 Department of Psychiatry, University of Campania “L. Vanvitelli”, Caserta, Italy; 4 Great Yarmouth Acute Service, Northgate Hospital, Norfolk & Suffolk NHS Foundation Trust, Great Yarmouth, UK; 5 Norwich Medical School, University of East Anglia, Norwich, UK

**Keywords:** coercion, Europe, involuntary treatment, mental health, psychiatry

## Abstract

Involuntary placement and treatment within mental healthcare represent one of the most sensitive areas where clinical needs and human rights intersect and the protection of fundamental rights of individuals subjected to coercive measures remains a paramount concern. This issue has been along-standing interest of the European Psychiatric Association (EPA), reflected in studies conducted by the EPA members and by the EPA Code of Ethics. Moreover, the EPA supports the work of the Parliamentary Assembly of the Council of Europe on a Draft Additional Protocol to the Convention on Human Rights and Biomedicine, as an European document aiming to harmonize practice across Europe, emphasizing involuntary treatment as a last resort, guaranteeing access to legal counsel, and ensuring continuous monitoring. However, weemphasize several aspects that should be included: 1) future reforms must integrate legalsafeguards with innovations in community care and evidence-based practice, ensuring that involuntary measures remain exceptional and rigorously justified; 2) while evidence-based strategies to reduce coercive treatment exist, it is important to emphasize the need for regular staff training, knowledge exchange, and consistent application of high standards, with a focus on minimizing the use of involuntary treatment within facilities while developing alternatives; 3)coercive treatment is regularly used in general hospitals for patients lacking decision-making capacity. Addressing all involuntary treatment, in both psychiatric and other healthcare settings, to ensure that the same legal, ethical and clinical values and standards are applied to all, is also critical in order to confine coercion to the absolute minimum.

## Context

Involuntary placement and treatment within mental healthcare represent one of the most sensitive areas where clinical necessity and human rights intersect. As mental health services continue to evolve, the protection of fundamental rights of individuals subjected to coercive measures remains a paramount concern. In European countries, clinical practices regarding involuntary placement and treatment are supported and defined by national legal frameworks that may vary across countries [1]. The Parliamentary Assembly of the Council of Europe is working on a Draft Additional Protocol to the Convention on Human Rights and Biomedicine, which deals with protecting the human rights and dignity of persons who are subject to involuntary placement and treatment within mental healthcare services. This underlines the urgency of standardizing safeguards that uphold dignity and autonomy while ensuring necessary care [2].

The European Psychiatric Association (EPA) represents over 78,000 psychiatrists across 47 national associations and 88 countries and actively contributes to shaping policies related to mental health care in Europe. At its 40th anniversary in December 2023, the EPA emphasized five priorities for mental health development through 2024–2029: harmonizing mental health care delivery, addressing workforce shortages, promoting ethical standards, innovating responses to evolving challenges, and fostering research and prevention [3]. The Manifesto was subsequently endorsed by GAMIAN and EUFAMI, forming a part of a collaborative Trialogue of Mental Health, involving clinicians, patients, and families, to promote policies on mental health in the European Union. These priorities directly touch on the issues surrounding involuntary treatment, especially given the association’s commitment to professional excellence and patient care.

## Human rights framework and ethical challenges

The protection of human rights in mental healthcare is underpinned by international legal instruments, such as the United Nations Convention on the Rights of Persons with Disabilities (CRPD), which emphasize autonomy, nondiscrimination, and the right to the highest attainable standard of health.

In its Code of Ethics (updated in 2024–2025), the EPA position on the use of involuntary (compulsory) measures is as follows: (1) the use of involuntary (compulsory) measures shall only be considered when all other options have been exhausted and no alternative is available to provide adequate care and ensure patient’s and/or other’s safety; (2) coercive measures should only be considered as a last resort; (3) when enforcing involuntary (compulsory) treatment, the psychiatrist shall comply with the laws in their respective country and cooperate with all personnel involved in this process; (4) involuntary (compulsory) care and treatment should only proceed while the patient continues to be a risk to themselves or others; (5) the patient’s status should be reviewed regularly with accordance to the relevant legal aspects in each European country that is represented in the EPA and consensus for treatment should be sought continuously; (6) even when patients lack competence to make treatment decisions as a result of psychiatric disorders, psychiatrists nonetheless keep them appropriately informed about their treatment and convey respect for their views; and (7) psychiatrists recognize that when patients regain competence, they can reassume their role as full partners in their psychiatric care” [4].

## Coercive practice in Europe

Despite these legal frameworks and ethical principles, coercive practices remain contentious. The 2017 report by the UN Special Rapporteur on the right to health highlighted ongoing human rights violations and called for noncoercive treatment models emphasizing prevention, social inclusion, and respect for dignity [5]. The report criticized the dominance of a biomedical model as a contributor to neglect and abuse in mental health practice, advocating for systemic reforms toward rights-based care, and stated that coercion is not justified, represents system failures, and should be abandoned. The report proposes actions to mainstream alternative policies, developing a roadmap to reduce coercion, exchanging good clinical practices, and investing in research, with a focus on prevention, service provision, and social aspects of mental health [5].

In response, the EPA recognized the importance of scientific evidence in interpreting the CRPD, advocating for shared decision-making paradigms while rejecting overly simplistic critiques of the biomedical model. The association emphasized that coercive treatment should remain a last-resort option, subject to rigorous scrutiny and balanced by adequately resourced, recovery-oriented alternatives to avoid harm to patients and others. However, the EPA emphasizes that complete elimination of all coercion, without adequately resourced, recovery-oriented noncoercive alternatives, would cause harm to service users and others. The EPA, therefore, calls for developing alternatives to long-term facilities for people in need rather than advocating abrupt closing down of facilities [6].

Across Europe, involuntary treatment is governed by diverse legal frameworks, yet common themes persist. In the first such study in 11 European countries (EUNOMIA), by Fiorillo et al. (2011), the authors analyzed similarities and differences of clinical conditions and legal prerequisites for involuntary hospital admission, professionals involved in involuntary hospital admission procedures, relationship with the patient and relatives, ethical aspects, and therapeutic plans [7]. They conclude that healthcare provided to patients should respect the principle of the “least restrictive alternative” and the relationship between patients and physicians should be based on reciprocal respect, because protection of patient civil rights and autonomy represents a fundamental goal of psychiatry [7].

In a more recent report on the status of involuntary treatment procedures in 40 European countries [1], the primary clinical justifications for involuntary admission remain risks of harm to self or others, severe self-neglect, and significant social functioning decline. However, the application of these criteria varies, reflecting different balances between medical and legal models and regional cultural factors. The authors recommended including medical aspects in the decision for involuntary treatment, where the need for medical treatment overrides social protection aspects [1]. In line with this recommendation, clinical examples may illustrate the need for involuntary interventions in situations such as acute delirium with aggression, intoxication with associated injury risk, severe psychosis with refusal of care, and dangerous behavioral disturbances. These cases highlight some of the complexities of ethical decision-making where patient capacity is impaired (Table 1).

**Table 1.** Clinical examples where some form of coercive treatment may be ethical

Clinical examples where some form of coercive treatment may be ethical
A patient with dementia who becomes physically aggressive toward family members due to disorientation and refuses any form of treatment.
An elderly patient in postoperative delirium after waking from general anesthesia.
A severely intoxicated young patient who has sustained life-threatening injuries and requires urgent medical attention.
A patient with severe mania or psychosis who, due to delusional thinking, is socially withdrawing, detaching from family and friends, refusing help, experiencing a significant decline in social functioning, and has stopped eating.
A patient with psychosis who denies that there is any problem, refuses treatment, and is driven by delusions exhibits serious aggression toward her children.
A very young child who is dying of septicemia but refuses treatment.
A person with a life-threatening condition, accompanied by severe confusion and who refuses treatment, such as diabetic ketoacidosis, a severe head injury, or severe intoxication.
An acutely suicidal person actively trying to hang themselves.

## Strategies to reduce coercion

To start, shared clinical decision-making is the predominant model in Europe, although there are differences between European regions [8]. Organization of psychiatric services following a community mental health model, such as flexible assertive community treatment (FACT) teams (e.g., Finland and the Netherlands), based on promotion of human rights, public health, recovery, effectiveness of interventions, community network, and peer support are thought to contribute to reducing involuntary placements. FACT teams that exemplify integrated, patient-centered care can mitigate crises and reduce coercion [9]. Although many intiatives exist to support the development of these services in the forms of EU project or professional networks (e.g., the European Community-based Mental Health Service Providers Network [https://eucoms.net/], the JA on Implementation of Best Practices in the Area of Mental Health [https://ja-implemental.eu/country-profiles-community-based-mental-healthcare-networks/], and the LaRge-scalE implementation of Community-based mental health care for people with seVere and Enduring mental ill health in EuRopE [https://horizoneurope.md/en/success-stories/recover-e-large-scale-implementation-community-based-mental-health-care-people]), community-based services are still developing in many European countries where hospital-based services remain the predominant model of care [10].

Regardless of the form of the mental health services, much improvement is possible as summarized in a recent document by the World Psychiatric Association [11]. Evidence-based interventions within existing inpatient settings have demonstrated reductions in coercive measures by 20–60% for some of the implemented measures, including staff education, environmental improvements, risk assessments, and post-incident debriefings [12, 13].

## Recommendations and future directions

We support the development of a European document, such as the proposed Additional Protocol to harmonize practice across Europe, emphasizing involuntary treatment as a last resort, guaranteeing access to legal counsel, and ensuring continuous monitoring. Future reforms must integrate legal safeguards with innovations in community care and evidence-based practice, ensuring that involuntary measures remain exceptional and rigorously justified. While evidence-based strategies to reduce coercive treatment exist, it is important to emphasize the need for regular staff training, knowledge exchange, and consistent application of high standards, with a focus on minimizing the use of involuntary treatment within facilities while developing alternatives.

Protecting the human rights of persons subjected to involuntary placement and treatment within mental healthcare services demands a delicate balance between safeguarding individual autonomy, protecting the right to life and health, and ensuring necessary care. A nuanced approach acknowledges the impaired decision-making capacity of some patients and the medical necessity of treatment beyond mere risk assessment. Upholding the right to proper medical care is a human right and not just an ethical imperative. It is foundational to the legitimacy and effectiveness of psychiatric care.

Coercive treatment is regularly used in general hospitals for patients lacking decision-making capacity, especially with children, and severely confused adults, and is therefore not just a mental health or a psychiatry issue. Addressing all involuntary treatment, in both psychiatric and other healthcare settings, to ensure that the same legal, ethical, and clinical values and standards are applied to all, is also critical to confine coercion to the absolute minimum.
